# Circadian Rhythms Time Seizure Severity in *Drosophila*

**DOI:** 10.1177/07487304261428337

**Published:** 2026-04-09

**Authors:** Mariam Huertas-Radi, Adam A. Bradlaugh, Richard A. Baines

**Affiliations:** * Division of Neuroscience, School of Biological Sciences, Faculty of Biology, Medicine and Health, University of Manchester, Manchester Academic Health Science Centre, Manchester, UK; † Institute of Neuro- and Behavioural Biology, Multiscale Imaging Centre, University of Münster, Münster, Germany

**Keywords:** *Drosophila*, circadian rhythms, seizure severity, bimodal rhythm, *period*, vortex assay

## Abstract

It is established that epilepsy patients can exhibit 24-h rhythms in seizure severity and occurrence. While the pathways underlying seizure rhythmicity remain poorly understood, it seems likely that a contribution from the biological clock is involved. A better understanding of any such contribution may translate to better treatments. Here, the influence of the 24-h circadian rhythm on seizure activity in *Drosophila melanogaster* is investigated. Seizure-susceptible bang-sensitive mutants (*julius seizure* and *paralytic^bangsenseless1^*) were subjected to mechanically induced seizure at 6 different zeitgeber points. A clear sex-dependent phenotype was observed, with seizure severity showing a greater time-of-day effect in females than males. The temporal pattern of seizure recovery time was bimodal, exhibiting both a morning and an evening peak. Rearing flies in constant light (LL), which renders the molecular clock dysfunctional, abolished the seizure rhythm. Conversely, female seizure mutants reared in constant darkness (DD), allowing free running of the circadian clock, continued to exhibit a bimodal rhythm of seizure severity. Moreover, seizure mutant females lacking a functional clock (*period^0^*) did not show rhythmicity of seizure severity. These findings support a role for the biological clock in seizure activity, at least in female *Drosophila*. Finally, seizure mutant females showed normal PERIOD clock protein intensity oscillations in clock neurons, supporting the hypothesis that seizure rhythmicity requires a functional circadian clock. Thus, this study validates *Drosophila* as a potential model for the identifying the mechanisms modulating seizure rhythmicity, with the potential to aid future treatment of epilepsy.

Epilepsy is a prevalent neurological disorder, affecting ~50 million people worldwide (WHO, 2024). Epilepsy is characterized by recurring seizures, the clinical manifestation of abnormal, excessive, and/or synchronous neuronal activity in the brain (Fisher et al., 2014). The most common treatment is antiseizure medications (ASMs), the large majority of which target ion channels or neurotransmitter signaling, acting to restore the excitatory-inhibitory balance (Löscher and Schmidt, 2011). However, despite more than 30 ASMs being developed, one third of people with epilepsy (PWE) remain drug refractory (Sultana et al., 2021). This underscores the need to identify novel targets and/or better ways to use existing ASMs.

Circadian rhythms are a fundamental aspect of biology, an internal timekeeping system that generates 24-h variations in most cellular, physiological, and behavioral processes. Circadian rhythms drive temporal patterns in processes critical for the initiation and spread of seizures (Meisel et al., 2015). Thus, the relevance of circadian rhythms to epilepsy, and the potential for treatment, is evident. Two of the most comprehensive databases of human seizures, SeizureTracker and NeuroVista, report 80% and 92% of PWE exhibiting seizures under circadian regulation, respectively (Karoly et al., 2018). Seizure occurrence and/or severity occur at fixed daily timings in most individuals. However, previous attempts at describing such temporal patterns have also uncovered significant variability (Leguia et al., 2021). Seizure rhythmicity is shown to depend on the brain region in which seizure onset occurs, seizure type, age, chronotype of patients, and sleep-wake cycles (Kaleyias et al., 2011; van Campen et al., 2015). Altogether, these factors reduce the accuracy of circadian rhythm evaluation in mammals and complicate the distinction of circadian rhythm effects on epilepsy from other influences.

The fruit fly, *Drosophila melanogaster*, provides a favorable model for studying circadian control of seizure severity (Johan Arief et al., 2018). Circadian rhythms in adult flies are orchestrated by ~240 clock neurons (Reinhard et al., 2024), compared with the human central circadian pacemaker suprachiasmatic nucleus with ~100,000 neurons (Swaab, 2003) and ~20,000 neurons in mice (Varadarajan et al., 2018). Despite the evolutionary distance, the *Drosophila* and human genomes are highly conserved, and molecular pathways implicated in human diseases can be modeled and studied in *Drosophila*, including epileptiform activity (Cunliffe et al., 2015). For example, *bang senseless* (*bss*) *Drosophila* mutants, carrying a gain-of-function mutation in the *paralytic* (*para*) gene coding for a voltage-gated sodium channel (Na_v_), exhibit seizures that resemble those of Na_v_-associated pharmacologically resistant epilepsies in humans (Parker et al., 2011). More specifically, Zhang et al. (2018) reported that exposing *Drosophila* to the ASM phenytoin during nighttime decreased seizure burden compared with daytime administration, reproducing the nighttime application of phenytoin to PWE that similarly decreased seizure burden and associated adverse effects (Yegnanarayan et al., 2006). Thus, the study of circadian rhythms contributing to seizure severity in fly seizure models has the potential to lead to significant progress in chronoepileptology.

Circadian rhythms are driven by clock genes, the proteins of which form a transcriptional-translational feedback loop that functions as a molecular clock. Clock gene expression is altered in human epileptogenic brain tissue (Li et al., 2017; Wu et al., 2021), and the manipulation of clock genes influences the rhythmicity of seizures in mouse models (Gerstner et al., 2014). Therefore, clock genes have been suggested to be important determinants of seizure activity. Despite the differences between the mammalian and fly molecular clock components, forward genetic screens have revealed a conserved mechanism that operates in key central pacemaker neurons to generate circadian rhythms (Hardin, 2011; Stanton et al., 2022). Ever since the first clock gene *period (per)* was cloned and characterized in *Drosophila*, this organism has been a model of choice for understanding how the molecular clock regulates rhythmic neuronal activity. Overall, *Drosophila* provides significant potential for the study of clock genes and neuronal excitability, involved in seizure initiation and propagation.

In this study, we explored the interplay between circadian rhythms and seizure severity in *Drosophila*, to provide evidence of the circadian clock contributing to seizure phenotype in fruit fly models of epilepsy. We report that bang-sensitive (BS) flies exhibit a bimodal circadian rhythm of seizure severity. This effect is larger and more pronounced in females. The influence of the circadian clock is abolished under constant light (LL) and in a *period null* (*per^0^*) mutation. Under constant darkness (DD) conditions, the time-of-day effect is still observable, as would be predicted if seizure activity is regulated by the molecular clock. As a result, our findings spotlight *Drosophila* as a model for identifying the mechanism(s) modulating seizure rhythmicity.

## Materials And Methods

### Fly Genetics and Husbandry

Flies were maintained on a standard cornmeal medium (5 L water, 390 g glucose, 360 g maize, 250 g yeast, 40 g agar, 135 mL nipagin, 15 mL propionic acid) at 25 °C under a 12:12-h light-dark (LD) cycle, unless otherwise stated. Canton-Special wild-type (CS), w^-^;+; *jus^iso7.8^* (referred to as *jus*), and *para^bss1^*; +; + (referred to as *para^bss1^*) stocks were obtained from the Baines lab. *per^0^*; +; *jus^iso7.8^* line (referred to as *per^0^*;;*jus*) was generated by M.H.R. in the Stanewsky lab.

### Lighting Conditions

Adult flies (0-16 h post-eclosion) were collected using CO_2_, separated by sex and placed into new food vials in groups of 6. Vials were either kept in a standard LD cycle or, when required, moved to LL or DD. Seizure induction and locomotor assays were performed under LD, LL, and DD. Light in LD was 1700 lux, and 1000 lux in LL, measured using a Thorlabs PM100A Power Meter.

### Seizure Induction: Vortex Assay

Seizure severity of 7 d old (±16 h) flies, of both sexes, was measured via vortex assays (Kuebler and Tanouye, 2000). Testing was conducted at zeitgeber times (ZT) 0, 4, 8, 12, 16 and 20; ZT 0 indicating the onset of the light phase and ZT 12 the onset of the dark phase. In constant conditions, flies were tested at circadian times (CT). Flies were kept in LL or DD for 7 days prior to testing. Testing was conducted within 1 h of each timepoint, equally spread across the 60 min for each lighting condition, genotype, and sex. To assess seizure severity in mutants and CS (used as controls), food vials sealed with flugs (packed cotton bungs) were placed upside down, such that flies stood on the flugs, and vortexed on a standard laboratory vortex mixer (Vortex Genie 2, Scientific Industries, USA) at maximum speed for 10 sec (Bermudez et al., 2023). Individual flies were only vortexed once, thus separate vials were vortexed at each ZT/CT. A total of 8 vials (6 flies per vial), per ZT, were vortexed for each genotype and sex. Seizure recovery time was measured as time required to regain posture and mobility for each fly. Seizure recovery time was used as a proxy for seizure severity; a higher recovery time indicated a more severe seizure.

### Locomotor Activity and Sleep: DAM System

Circadian locomotor activity and sleep of female flies was measured using the Drosophila Activity Monitor (DAM) 5M software (TriKinetics Inc., Waltham, USA). Seizure mutant *jus* female flies (and CS used as controls) were collected upon eclosion (±16 h), anesthetized with CO_2_, and transferred individually into DAM tubes (5 mm diameter, 65 mm length) using a fine paintbrush. Tubes were prepared by adding ~5 mm standard cornmeal medium (see above) to one end of the tube, sealed with a rubber cap, and a small cotton plug to the other end. Before any recordings started, flies were allowed 24 h to adapt to the new tube. DAM monitors with 32 flies per genotype, and per lighting condition, were initially entrained for 3 days in LD, followed by 5 days of either DD or LL. Experiments were run at 25° C, and an environmental monitor (DEnM; Trikinetics Inc.) was used to monitor conditions. Locomotor activity of flies was summed into 1-min bins. Flies that died before the end of the experiment were excluded from analysis. In all experiments, data are from flies collected and run concurrently, per lighting condition. DAM circadian and sleep data were analyzed using the Sleep and Circadian Analysis Matlab Program (SCAMP, MATLAB 24.2) (Vecsey et al., 2024).

### Immunostaining and Confocal Microscopy

Flies (0-1 day old) entrained to LD through the final 5 days of development were collected in the appropriate lighting condition and fixed at ZT 10 and ZT 22 with 4% paraformaldehyde in 0.1% PBST for 2.5 h at room temperature. Following 6 washes of 10 min each in 0.1% PBST, brains were dissected in 0.1% PBST and blocked for 2 h at room temperature in 5% goat serum (0.1% PBST). Primary antibodies were added and incubated for 48 h (in 5% goat serum in 0.1% PBST) at 4 °C. Mouse anti-PDF (DSHB, AB-760350) was used at 1:500, and rabbit anti-PERIOD (Stanewsky lab) at 1:5000. Following 6 washes of 10 min each in 0.1% PBST, secondary antibodies were added and incubated overnight (in 5% NGS in 0.1% PBST) at 4 °C. Goat anti-mouse AlexaFluor 647 nm (Thermo Fisher Invitrogen, A-21235) was used at 1:500, and goat anti-rabbit AlexaFluor 488 nm (Thermo Fisher Invitrogen, A-11008) at 1:500. Following 6 washes of 10 min each in 0.1% PBST, brains were mounted using Vectashield antifade mounting medium (Vector Laboratories, H-1700-10). Brains were imaged with a Leica TCS SP8 confocal microscope with a 40× objective. Average intensity was measured using (Fiji Is Just) ImageJ (1.54p), and quantification was normalized to the background: (PER signal − background signal) / area.

### Statistical Analysis

**Figure 1.**
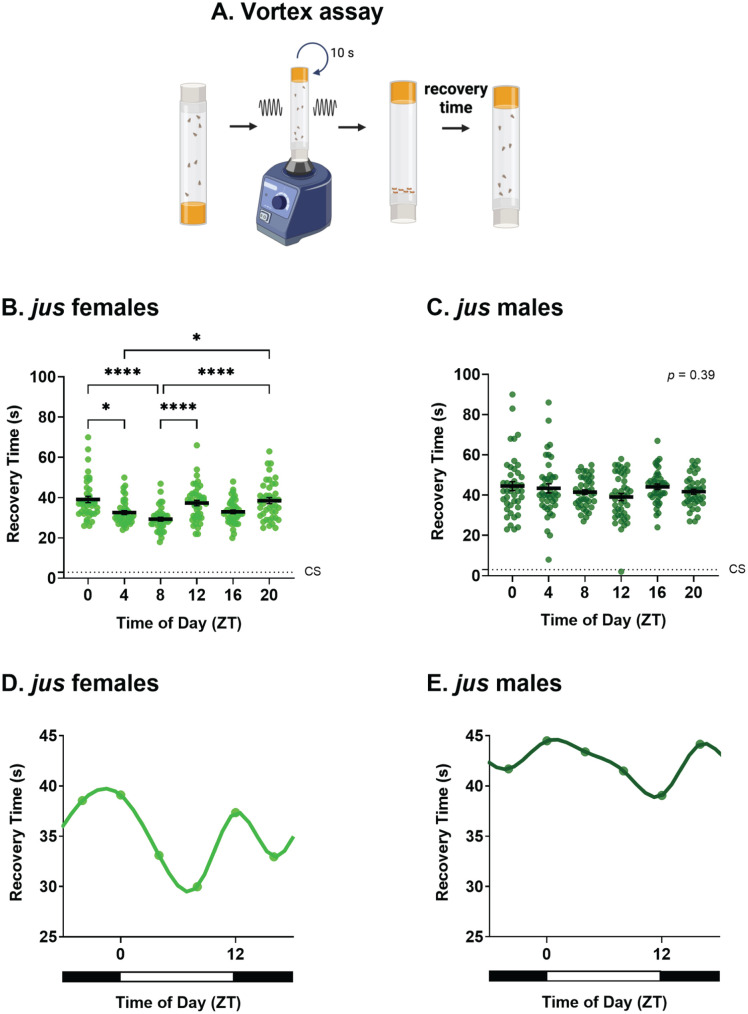
Effect of zeitgeber time (ZT) on seizure recovery time of *jus* seizure mutants. Recovery time (s) of adult flies in 12:12 light-dark conditions, tested via the vortex assay at ZT 0, 4, 8, 12, 16, and 20. (a) Schematic of the vortex assay. Created in BioRender. Huertas Radi, M. (2025) (https://BioRender.com/u6v9tc9. (b) *jus* females show a statistically significant effect of ZT on seizure recovery time, Kruskal-Wallis test, *H*(5) = 43.92, *p* < .0001, *n* = 39-43. (c) *jus* males show no statistically significant effect of ZT on recovery time, Kruskal-Wallis test, *H*(5) = 5.23, *p* = .39, *n* = 41-43. Average seizure recovery time of wild-type CS controls is shown by dotted lines and are significantly different than *jus* female and male mutants, Kruskal-Wallis test, *H*(6) = 109.0 (females), 84.65 (males), *p* < .0001 for both. (d) Circadian profile of seizure severity of female *jus* exhibits a bimodal circadian rhythm. (e) Circadian profile of seizure severity of male *jus* exhibits a similar phase to females but with a smaller effect size. For b and c, individual dots are recovery times of individual flies vortexed only once, derived from a total of 8 vials (6 flies per vial), per ZT and sex. Statistically significant comparisons from Dunn’s multiple comparison tests are indicated as **** *p* ≤ .0001, *** *p* ≤ .001, ** *p* ≤ .01, * *p* ≤ .05. Full descriptive statistics and *p* values are reported in Table 1. Data are presented as mean ± SEM. For d and e, data are presented as means of b and c vortex data, respectively, for each ZT. Horizontal white and black bars indicate when lights were on (ZT 0 to ZT 12) and off (ZT 12 to ZT 24).

**Figure 2.**
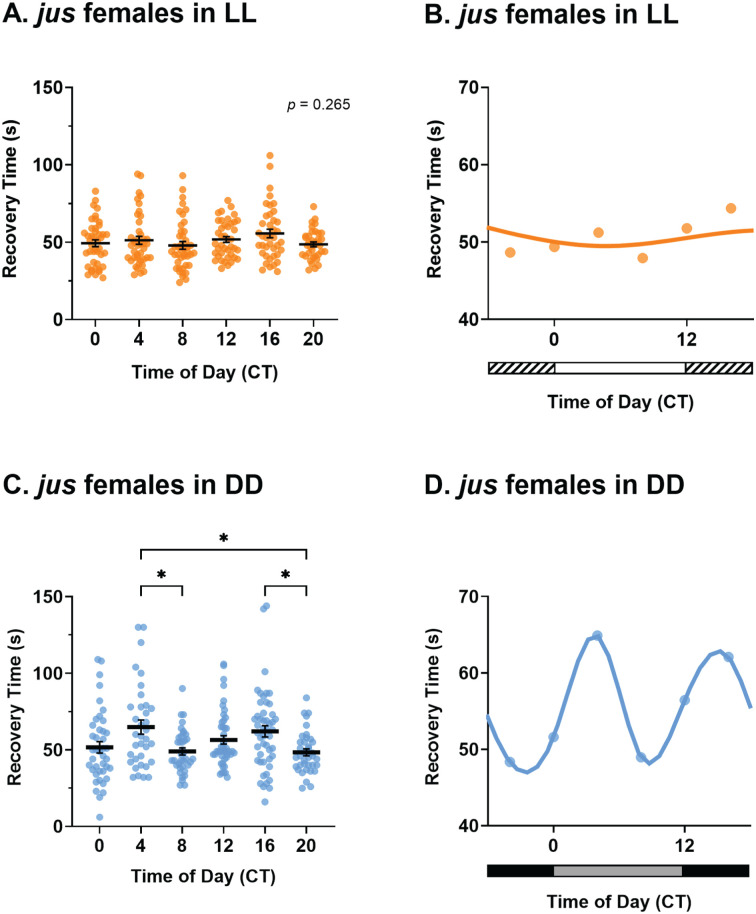
Rhythmicity of *jus* female seizure severity is abolished in constant light (LL) but maintained in constant darkness (DD). (a) Seizure recovery times (s) of *jus* females tested via the vortex assay after 7 days of LL show no effect of circadian time (CT), Kruskal-Wallis test, *H*(5) = 6.45, *p* = .26, *n* = 37-43. (b) Temporal pattern of seizure severity of *jus* females, reared in LL, shows no obvious rhythmicity. (c) *jus* females under DD show a significant effect of CT on seizure recovery time, Welch’s ANOVA, *W*(5, 108.92) = 4.23, *p* < .01, *n* = 35-49. (d) Circadian profile of seizure severity of *jus* females reared in DD exhibits a bimodal circadian rhythm. For a and c, individual dots are recovery times of individual flies vortexed only once, derived from a total of 8 vials (6 flies per vial), per CT and lighting condition. Statistically significant comparisons from Dunnett’s multiple comparison tests are indicated as *p ≤ .05. Full descriptive statistics and *p* values are reported in Table 1. Data are presented as mean ± SEM. For b and d, data are presented as means of a and c, respectively. In b, horizontal white and striped bars represent periods of light and “subjective nighttime” in LL, respectively. In d, horizontal black and gray bars represent periods of dark and “subjective daytime” in DD, respectively.

Statistics were performed in GraphPad Prism (10.4.1). All data obtained were tested for normality and equal variances using Shapiro-Wilk and Brown-Forsythe tests, respectively, before any further statistical analyses were carried out. Sample sizes for vortex assays were determined from previous studies (Mituzaite et al., 2021). Outlier tests were performed on vortex assay data to remove obvious outliers following ROUT 1% criteria. For Figures 1b, 1c, 2a, 2c, 5a, 6a, and 6c, 1-way analysis of variance (ANOVA) (or the equivalent nonparametric Kruskal-Wallis test, or Welch’s ANOVA for unequal variances) was used to determine the effect of ZT or CT on seizure recovery time. For Figures 1d, 1e, 2b, 2d, 5b, 6b, and 6d, cubic spline curves were generated from means to visualize circadian rhythms in seizure severity. Flies were considered rhythmic when the calculated rhythmicity strength (RS) ≥ 2. For Figure 4a to 4d and Supplemental Figure S2A to D, 1-way ANOVA (or the equivalent non-parametric Kruskal-Wallis test) was used to determine differences in PER intensity in clock neurons between ZTs. For Supplemental Figure S1A to 1D, 1-way ANOVA (or the equivalent non-parametric Kruskal-Wallis test, or Welch’s ANOVA for unequal variances) was performed to compare circadian locomotor activity and sleep data obtained from SCAMP between lighting conditions. The level of significance accepted for all tests was *p* ≤ .05. For significant test results, the appropriate post hoc test was performed.

## Results

### Circadian Bimodal Pattern of Seizure Recovery Time in Females

The aim of this study was to establish the influence of circadian timing on seizure severity in *Drosophila*. To address this, BS seizure-susceptible *julius seizure* (*jus*) mutants were used. These mutants are defective for an uncharacterized protein-coding gene *CG14509* (Horne et al., 2017) and show prolonged seizures when subjected to a strong mechanical shock that simultaneously activates sensory input from peripheral hairs. However, this mutation does not show spontaneous seizures. Male and female *jus* flies, under 12:12 LD conditions, were subjected to vortex assays to induce seizures at ZT 0, 4, 8, 12, 16, and 20, and recovery times measured (Figure 1a). Seizure recovery time in our assay was measured as the time taken for flies to recover posture and mobility, thus including any post-ictal refractory period during which a fly may not be seizing, but is still immobile. The recovery time of CS, measured at the same time, was significantly lower than *jus* mutants, independent of sex (Kruskal-Wallis tests, *p* < .0001), thus validating the seizure phenotype observed.

Recovery time from induced seizure in *jus* showed a sex-dependent bias. Here, *jus* females showed a statistically significant effect of ZT on seizure recovery (Figure 1b, Kruskal-Wallis test, *p* < .0001). Dunn’s multiple comparison tests revealed significant differences between ZT 0-4, ZT 0-8, ZT 4-20, ZT 8-12, and ZT 8-20 (full descriptive statistics and *p* values are reported in Table 1). By contrast, males showed no statistically significant differences in recovery time (i.e., seizure severity) across timepoints (Figure 1c, Kruskal-Wallis test, *p* = .39). Interestingly, *jus* males showed increased recovery times across the circadian cycle compared with females. A 2-way ANOVA showed a significant effect of sex on seizure recovery times (2-way ANOVA, *p* < .0001).

**Table 1.** Full descriptive statistics of multiple comparison tests of seizure severity between timepoints of *jus* females.

Condition and Genotype	Timepoints Compared	Mean Recovery Time (s)	SEM	Post Hoc Test	*p* Value	Significance	Figure
LD *jus*	ZT 0 ZT 4	39.10 32.58	1.57 0.96	Dunn’s	<.05	*	1b
	ZT 0 ZT 8	39.10 29.33	1.57 0.94	Dunn’s	<.0001	****	1b
	ZT 4 ZT 20	32.58 38.54	0.96 1.48	Dunn’s	<.05	*	1b
	ZT 8 ZT 12	29.33 37.35	0.94 1.35	Dunn’s	<.0001	****	1b
	ZT 8 ZT 20	29.33 38.54	0.94 1.48	Dunn’s	<.0001	****	1b
DD *jus*	CT 4 CT 8	64.89 48.95	4.62 2.32	Dunnett’s	<.05	*	2c
	CT 4 CT 20	64.89 48.35	4.62 2.24	Dunnett’s	<.05	*	2c
	CT 16 CT 20	62.08 48.35	3.71 2.24	Dunnett’s	<.05	*	2c
DD *para^bss1^*	CT 0 CT 8	786.27 544.33	21.91 31.78	Dunn’s	<.0001	****	5c
	CT 0 CT 12	786.27 594.79	21.91 25.55	Dunn’s	.0001	***	5c
	CT 0 CT 16	786.27 519.33	21.91 35.92	Dunn’s	<.0001	****	5c
	CT 0 CT 20	786.27 466.65	21.91 22.33	Dunn’s	<.0001	****	5c
	CT 4 CT 8	691.96 544.33	23.7 31.78	Dunn’s	.014	*	5c
	CT 4 CT 16	691.96 519.33	23.7 35.92	Dunn’s	.0003	***	5c
	CT 4 CT 20	691.96 466.65	23.7 22.33	Dunn’s	<.0001	****	5c
	CT 12 CT 20	594.79 466.65	25.55 22.33	Dunn’s	.022	*	5c

To further examine the temporal pattern of *jus* seizure severity, spline curves were generated from vortex assay data. In females, cycles of seizure recovery exhibited a bimodal rhythm, exhibiting 2 peaks (ZT = 12.5 h and ZT = 22.7 h) and 2 troughs (ZT = 6.9 h and ZT = 16.2 h) across the 24 h day (Figure 1d). Notably, spline curves generated for males also showed a similar temporal pattern, however, with a much smaller effect size (Figure 1e). Therefore, we conclude that seizure severity tested by the vortex assay is influenced by ZT, with an effect size that is larger in female *jus* mutants.

### Circadian Rhythm Abolished in Constant Light but Maintained in Constant Darkness

To provide additional evidence for a contribution of CT to seizure recovery, we determined the effects of altering the molecular clock. Under conditions of constant light (LL), *Drosophila* are arrhythmic because of continuous degradation of the protein TIMELESS (TIM) by its interaction with CRYPTOCHROME (CRY) (Marrus et al., 1996). Thus, the expectation, in keeping with a contribution from the molecular clock, is that under LL a bimodal pattern of seizure severity would be absent. We focused attention on *jus* females, given the larger effect size. Indeed, *jus* females reared under LL showed no significant difference in recovery times between CT (Figure 2a and 2b, Kruskal-Wallis test, *p* = .26). Therefore, time of day did not influence seizure severity of *jus* females in LL.

Under conditions of constant darkness (DD), the *Drosophila* molecular clock free-runs (Konopka and Benzer, 1971). Maintenance of a bimodal rhythm of seizure severity under DD would, thus, further support our hypothesis of a contribution from the molecular clock to seizure severity, rather than simply being driven by the light-dark cycle. Indeed, seizure recovery times of *jus* females kept in DD were significantly influenced by CT (Figure 2c and 2d, Welch’s ANOVA, *p* < .01). Dunnett’s multiple comparison tests showed significant differences between CT 4-8, CT 4-20, and CT 16-20 (full descriptive statistics and *p* values are reported in Table 1). Therefore, time of day influenced seizure severity of *jus* females in DD. The temporal pattern of seizure severity was circadian, exhibiting 2 peaks (CT = 4.1 h and CT = 15.3 h) and 2 troughs (CT = 8.7 h and CT = 21.8 h) across the 24 h day (Figure 2d). The timing of peaks and troughs, and therefore the phase of the DD cycle, was shifted to ~4 h later relative to the LD cycle. Taken together, these results validate a hypothesis that the circadian clock governs seizure severity in *jus* females.

Independent of CT, *jus* females in LL and DD showed an increase in seizure severity (i.e., a longer recovery time) compared with LD (Supplemental Figure S1A and Table S1, 2-way ANOVA, *p* < .0001). Sleep characteristics of *jus* females were monitored via the TriKinetics system, showing reduced total amount of sleep and shorter duration of sleep bouts of *jus* females reared in LL and DD conditions, compared with LD. The number of sleep episodes was also increased under LL but remained unchanged under DD (Supplemental Figure S1B-S1D and Table S2). Preclinical and clinical studies have shown that sleep deprivation can increase the risk of epileptic seizures in humans (Bonilla-Jaime et al., 2021; Dell’Aquila and Soti, 2022). Potentially, sleep disturbances experienced by *jus* females in constant conditions could increase seizure severity when tested via the vortex assay. These data further support the potential *Drosophila* holds as a seizure model to study circadian and sleep behavior.

### Circadian Locomotor Activity of *jus* Mutants Complements Seizure Rhythmicity Findings

**Figure 3.**
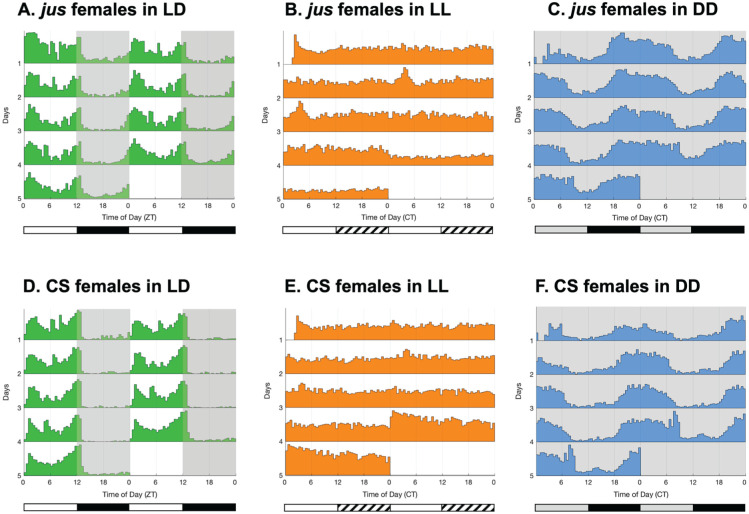
Double-plotted actograms of circadian locomotor activity of *jus* and CS females in 12:12 light-dark (LD), constant light (LL), and constant darkness (DD) conditions. Activity of adult females was analyzed via the TriKinetics system. (a) Locomotor activity of *jus* females in 12:12 LD shows a bimodal circadian rhythm, with peaks of activity at ZT 0 and ZT 12 (*n* = 28). (b) Locomotor activity of *jus* females in LL (switched to LL after 3 days of LD) shows arrhythmicity, with a constant level of activity lacking any temporal pattern (*n* = 25). (c) Locomotor activity of *jus* females in DD (switched to DD after 3 days of LD) shows a circadian rhythm, but not bimodal due to the lack of a “siesta” period (*n* = 27). (d) Locomotor activity of CS females in 12:12 LD shows a bimodal circadian rhythm, with peaks of activity at ZT 0 and ZT 12 (*n* = 16). (e) Locomotor activity of CS females in LL (switched to LL after 3 days of LD) shows arrhythmicity, with a constant level of activity lacking any temporal pattern (*n* = 26). (f) Locomotor activity of CS females in DD (switched to DD after 3 days of LD) shows a circadian rhythm, but not bimodal due to the lack of a “siesta” period (*n* = 24). In actograms, lights-off are indicated by gray background shading, and lights-on are indicated by white shading. Horizontal white and black bars under LD actograms indicate when the lights were on (ZT 0 to ZT 12) and off (ZT 12 to ZT 24). Horizontal white and striped bars under LL actograms represent periods of light and “subjective nighttime,” respectively. Horizontal black and gray bars under DD actograms represent periods of dark and “subjective daytime,” respectively.

To further support the possible contribution of the circadian clock to seizure activity, locomotor activity of *jus* females was measured via the TriKinetics system under the same lighting conditions as vortex assays (Figure 3a-3c). As controls, CS female flies were monitored under the same conditions (Figure 3d-3f). In LD, adult *jus* females showed an expected bimodal circadian rhythm of locomotion, exhibiting morning and evening peaks of activity at ZT 0 and ZT 12 (Figure 3a), similar to CS females (Figure 3d). Conversely, when kept in LL conditions, *jus* females did not show rhythmicity or any pattern of locomotor activity (Figure 3b), similar to CS females (Figure 3e). Finally, when kept under free-running DD conditions, *jus* females showed circadian rhythmicity (Figure 3c), similar to CS females (Figure 3f). Thus, *jus* females exhibit behavior consistent with a functional clock and in line with control CS flies. It is worth noting that the activity profile of *Drosophila* in DD does not exhibit 2 distinctive peaks of morning and evening activity as in LD, but rather one merged peak of activity. Note that fly lines used for activity monitoring were not isogenic lines; we therefore focused on environmental manipulations, such as LL or DD exposure; thus, meaningful comparisons can only be made within genotypes between conditions.

Values regarding rhythmicity, period length, RS, and RI are reported in Table 2. Comparisons of rhythmicity values of *jus* females across lighting conditions revealed that the RS and RI values in LL were significantly lower than in LD and DD (Kruskal-Wallis with Dunn’s multiple comparison tests, *p* < .0001, for both). No significant differences were found between rhythmicity values in LD and DD (Kruskal-Wallis with Dunn’s multiple comparison test, *p* = .85). Therefore, this pattern of circadian locomotor activity validates that, as expected, the behavior of *jus* females in LD and DD conditions is rhythmic, with a period of around 24 h, while arrhythmic in LL, complementing the loss of seizure severity rhythms when *jus* females were reared in LL prior to vortex testing.

**Table 2.** Rhythmicity percentages, period length, rhythmicity strength (RS) and rhythmicity index (RI) of CS and *jus* female flies in different lighting conditions.

Genotype	Lighting Condition	Rhythmicity	Period	RS	RI
CS	LD	100%	24.03 ± 0.03	3.43 ± 0.17	0.44 ± 0.02
*jus*	LD	85.71%	23.95 ± 0.03	2.82 ± 0.21	0.36 ± 0.03
CS	LL	11.54%	-	0.65 ± 0.2	0.08 ± 0.02
*jus*	LL	4%	-	0.72 ± 0.13	0.09 ± 0.02
CS	DD	87.5%	24.64 ± 0.22	2.52 ± 0.18	0.32 ± 0.02
*jus*	DD	88.89%	24.15 ± 0.06	2.61 ± 0.15	0.33 ± 0.02

### *jus* Female Mutants Show Normal PER Oscillations in Clock Neurons

**Figure 4.**
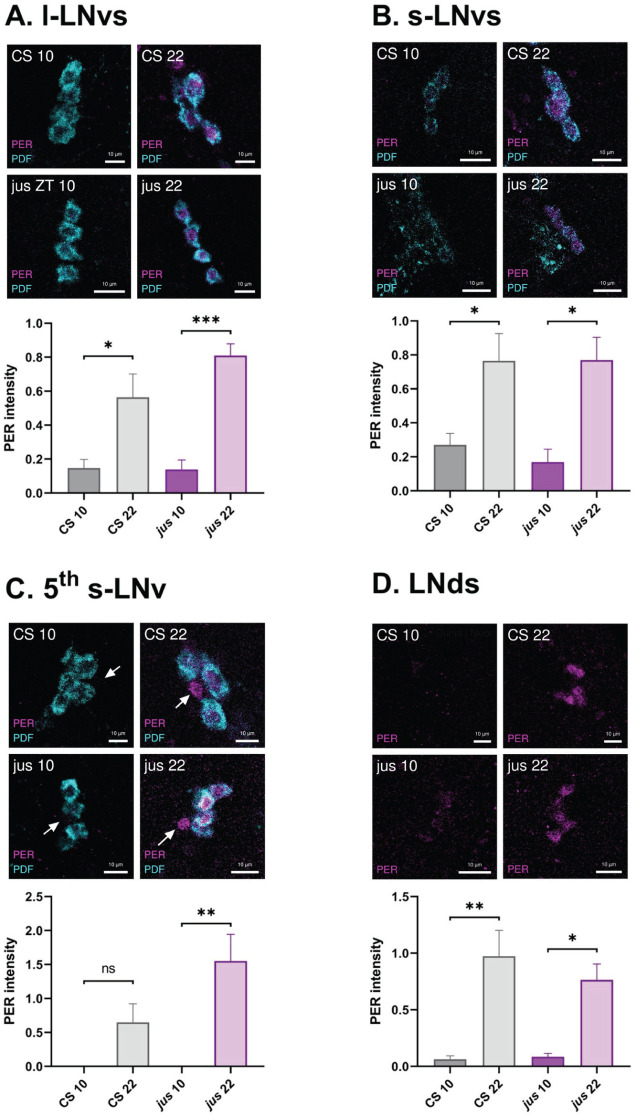
*jus* females show PER oscillations in clock neurons. The intensity of PER expression was quantified from antibody stainings in *jus* and CS control clock neurons: large ventral-lateral neurons (l-LNvs, a), small ventral-lateral neurons (s-LNvs, b), the 5th s-LNv (c), and dorsal-lateral neurons (LNds, d). Representative images of PER (in magenta) and PDF (in cyan) immunostainings of CS and *jus* females at ZT 10 and ZT 22 are displayed above the respective quantifications. PDF was stained for accurate localization of l-LNvs and s-LNvs. Scale bars, 10 μm. Both CS (in gray) and *jus* (in purple) female flies showed statistically significant differences between PER intensity at ZT 10 and 22 in (a) l-LNvs (1-way ANOVA with Tukey’s multiple comparison test, *p* *<* .05 and *p* < .001, respectively); (b) s-LNvs (1-way ANOVA with Tukey’s multiple comparison test, *p* < .05 and *p* < .05, respectively); (d) LNds (1-way ANOVA with Tukey’s multiple comparison test, *p* < .01 and *p* < .05, respectively). For the 5th s-LNv (c), only *jus* females showed a statistically significant difference, whereas for CS it was nonsignificant (1-way ANOVA with Tukey’s multiple comparison test, *p* < .01 and *p* = .26, respectively). Statistically significant comparisons from Tukey’s multiple comparison tests are indicated as *** *p* ≤ .001, ** *p* ≤ .01, * *p* ≤ .05. Clock neurons from 5 brain hemispheres were quantified for each condition. Quantification was normalized to the background. Data are presented as mean ± SEM.

To confirm that the core molecular oscillator is intact in the *jus* background, PER immunostainings were performed at ZT 10 and ZT 22 to compare PER expression in 4 clock neuron groups: the large ventral-lateral neurons (l-LNvs, Figure 4a), the small ventral-lateral neurons (s-LNvs, Figure 4b), the 5th s-LNv (Figure 4c), and the dorsal-lateral neurons (LNds, Figure 4d). As expected, *jus* females showed higher PER intensity at ZT 22 than ZT 10 in all clock neuron groups measured (1-way ANOVA with Tukey’s multiple comparisons, *p* < .001, *p* < .05, *p* < .01, *p* < .05, respectively). Interestingly, *jus* males did not show a statistically significant difference in PER intensity between timepoints in any of the groups, although the data were trending (Supplemental Figure S2).

For comparison, female CS adult brains were also immunostained for PER at ZT 10 and ZT 22. As expected, PER staining in the l-LNvs (Figure 4a), s-LNvs (Figure 4b), and LNds (Figure 4d) showed higher intensity at ZT 22 than ZT 10 (1-way ANOVA with Tukey’s multiple comparisons, *p* < .05, *p* < .05, *p* < .01, respectively). In the 5th s-LNv (Figure 4c), CS females did not show a significant difference in PER intensity between timepoints (1-way ANOVA with Tukey’s multiple comparisons, *p* = .26), although the data were trending. CS males also showed significantly higher PER intensity at ZT 22 than ZT 10 in the l-LNvs, the 5th s-LNv, and the LNds, but not in the s-LNvs (Supplemental Figure S2).

Comparing CS control females to seizure mutant *jus* females, PER intensity values were not significantly different between genotypes, in any of the 4 clock neuron groups at the 2 timepoints measured (*p* values are reported in Table 3). Taken together, PER expression supports a hypothesis that seizure rhythmicity in *jus* females emerges from a functional clock.

**Table 3.** Statistical comparison of PER intensity of CS (controls) and *jus* females at ZT 10 and 22, in 4 clock neuron groups.

Genotypes Compared	Timepoint	Clock Neuron Group	Post Hoc Test	*p* Value	Significance
CS and *jus*	ZT 10	l-LNvs	Tukey’s	=.99	ns
		s-LNvs	Tukey’s	=.93	ns
		5th s-LNv	Tukey’s	>.99	ns
		LNds	Tukey’s	=.99	ns
CS and *jus*	ZT 22	l-LNvs	Tukey’s	=.22	ns
		s-LNvs	Tukey’s	>.99	ns
		5th s-LNv	Tukey’s	=.07	ns
		LNds	Tukey’s	=.7	ns

### *jus* Female Seizure Rhythmicity Requires *period*

**Figure 5.**
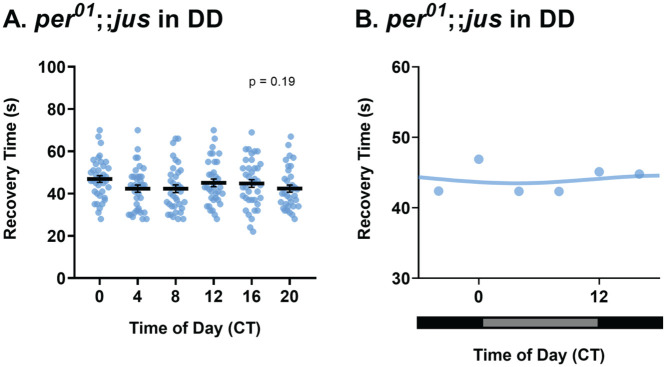
Rhythmicity of seizure severity is abolished in *per^0^*;;*jus* females. (a) Seizure recovery time (s) of *per^0^*;;*jus* females tested via the vortex assay after 3-4 days in LD, followed by 7 days of DD show no effect of circadian time (CT), Kruskal-Wallis test, *H*(5) = 7.37, *p* = .19, *n* = 34-39. Individual dots are recovery times of individual flies vortexed only once, derived from a total of 8 vials (6 flies per vial), per CT. Data are presented as mean ± SEM. (b) Temporal pattern of seizure severity of *jus* females, reared in DD after 3 days of LD, shows no obvious rhythmicity. Data are presented as means of a. Horizontal black and gray bars represent periods of dark and “subjective daytime” in DD, respectively.

While manipulating lighting conditions provides evidence that seizure severity is regulated by the circadian clock, we also combined a loss-of-function *per^0^* mutation with the *jus* seizure mutant, generating a *per^0^*;;*jus* line. The *per^0^* mutation renders flies arrhythmic in DD (Wheeler et al., 1993); thus, seizure mutants with this mutation would be expected to not exhibit a rhythm of seizure severity. Indeed, *per^0^*;;*jus* females raised in LD and exposed to 7 days DD before vortex testing showed no significant difference in seizure recovery times between CTs (Figure 5a and 5b, Kruskal-Wallis test, *p* = .19). Therefore, time of day did not influence seizure severity of *per^0^*;;*jus* females in DD. This observation provides strong support for seizure rhythmicity in *jus* females being driven by the core clock, as *per* is required for seizure severity rhythms of *jus* females.

### Circadian Modulation of Seizure Recovery Time Is a Broad Phenomenon Across *Drosophila* Seizure Mutants

**Figure 6.**
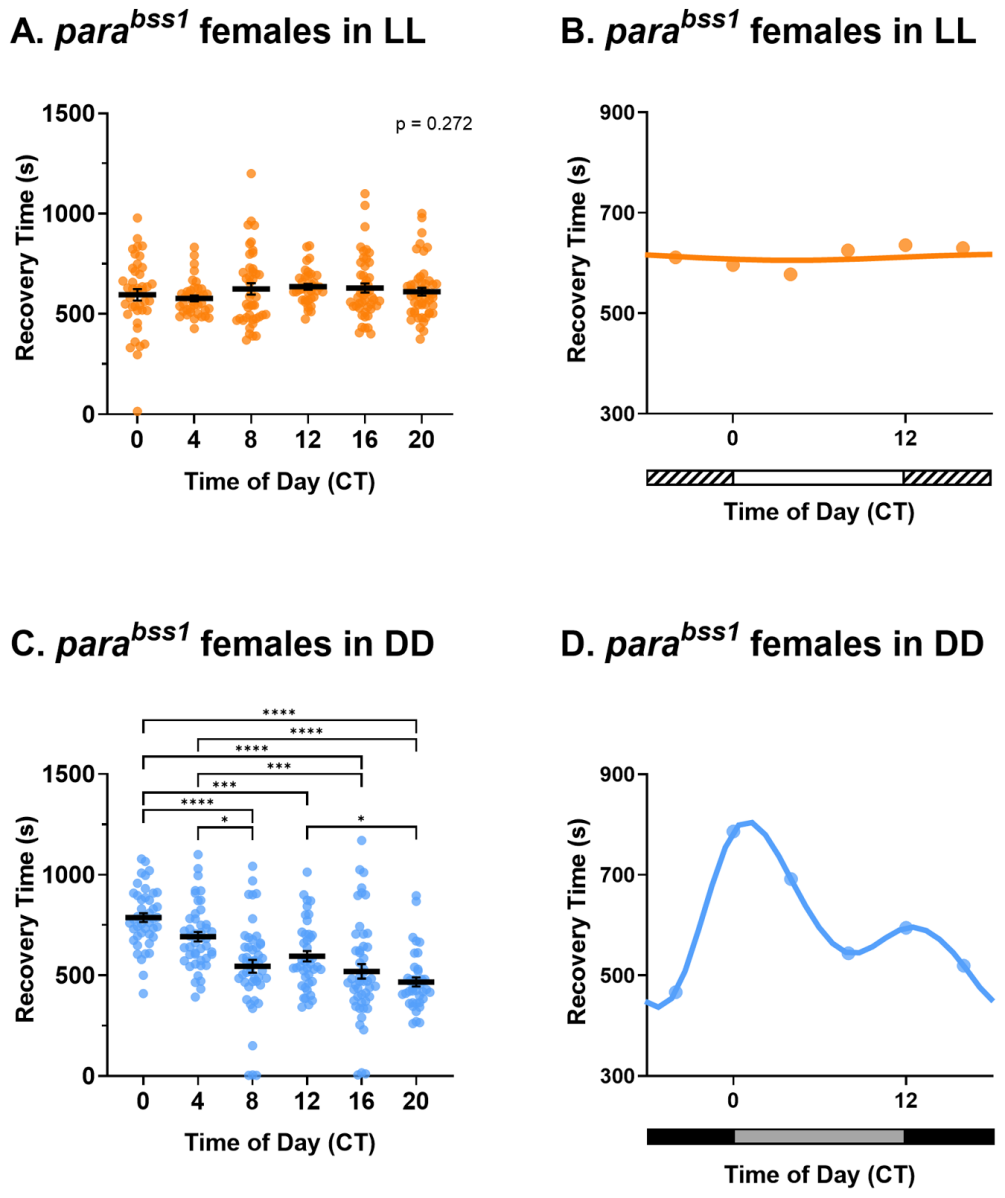
*para^bss1^* females show rhythmicity of seizure recovery in constant darkness (DD), but not in constant light (LL). (a) Seizure recovery times (s) of *para^bss1^* females tested via the vortex assay after 7 days of LL show no effect of circadian time (CT), Kruskal-Wallis test, *H*(5) = 6.37, *p* = .27, *n* = 36-50. (b) Temporal pattern of seizure severity of *para^bss1^* females, reared in LL, shows no obvious rhythmicity. (c) *para^bss1^* females under DD show a significant effect of CT on seizure recovery time, Kruskal-Wallis test, *H*(5) = 80.66, *p* < .0001, *n* = 40-49. (d) Circadian profile of seizure severity of *para^bss1^* females reared in DD exhibits a bimodal circadian rhythm. For a and c, individual dots are recovery times of individual flies vortexed only once, derived from a total of 8 vials (6 flies per vial), per CT, and lighting condition. Statistically significant comparisons from Dunn’s multiple comparison tests are indicated as **** *p* ≤ .0001, *** *p* ≤ .001, ** *p* ≤ .01, *p ≤ .05. Full descriptive statistics and *p* values are reported in Table 1. Data are presented as mean ± SEM. For b and d, data are presented as means of a and c, respectively. In b, horizontal white and striped bars represent periods of light and “subjective nighttime” in LL, respectively. In D, horizontal black and gray bars represent periods of dark and “subjective daytime” in DD, respectively.

To further validate circadian effects observed on seizure recovery time of *jus* female mutants, a different BS mutant was tested. *Para^bangsenseless1^* (*para^bss1^*) is a gain-of-function mutation in the sole voltage-gated sodium channel (Na_v_) in *Drosophila* (Parker et al., 2011). These mutants exhibit a more severe seizure phenotype compared with *jus* when subjected to strong mechanical shock, reflected in their recovery time (Mituzaite et al., 2021). Female *para^bss1^* flies reared in LL and DD were subjected to vortex assays using the same methodology as *jus* in Figure 2. Recovery times of *para^bss1^* females in LL conditions were not different across timepoints (Figure 6a and 6b, Kruskal-Wallis test, *p* = .272). Therefore, time of day did not influence seizure severity of *para^bss1^* females in LL. By contrast, recovery times of *para^bss1^* females in DD, with a free-running clock, were significantly influenced by CT (Figure 6c, Kruskal-Wallis test, *p* < .0001). Dunn’s multiple comparisons test showed significant differences between recovery times at CT 0-8, CT 0-12, CT 0-16, CT 0-20, CT 4-8, CT 4-16, CT 4-20, and CT 12-20 (full descriptive statistics and *p* values are reported in Table 1). Therefore, time of day influenced seizure severity of *para^bss1^* females in free-running conditions. The temporal pattern of seizure severity was circadian, exhibiting 2 peaks (CT = 1.3 h and CT = 12.5 h) and 2 troughs (CT = 8.7 h and CT = 18.8 h) across the 24 h day (Figure 6d). Taken together, these results validate the hypothesis that the circadian clock governs seizure severity in females, and that this is not specific to *jus*, but a phenomenon across at least 2 different *Drosophila* seizure mutants.

## Discussion

The aim of this study was to determine whether circadian timing influences *Drosophila* seizure severity. To achieve this, seizure recovery time of *jus* mutants were recorded at different times of the day. Male flies showed no effect of time on seizure severity, whereas females revealed an influence of ZT on seizure severity measured via the vortex assay. In humans, sex differences are evident in many epilepsies and seizure conditions (Hophing et al., 2022). Neurotransmission and synaptic function, which mediate neural activity, and thus seizure activity, are modulated in a circadian-dependent and sex-specific manner (Logan et al., 2022). Current evidence suggests that males exhibit greater overall susceptibility to excitability episodes and occurrence of seizures, while females exhibit greater fluctuations in seizure occurrence (Reddy et al., 2021). The precise molecular mechanisms of this remain unknown, although most studies suggest the involvement of neuroendocrine factors (Christian et al., 2020). The only sex differences reported in *Drosophila* relating to epilepsy are at the transcriptomic level in pentylenetetrazol-induced seizure models, where males showed multiple developmental signaling pathways downregulated, and females only showed downregulation of genes related to the ribosomal pathway, suggesting reduced translational capacity (Sharma et al., 2009). Thus, behavioral differences between males and females in seizure rhythmicity have not been previously reported. Given the sex differences observed in this study, future work on *Drosophila* male and female hormones could perhaps offer an opportunity to elucidate the mechanisms underlying sex differences in epilepsy.

In *Drosophila*, mating status is known to influence behavior (Kubli, 2003). Mating affects sleep (Geissmann et al., 2019) and circadian behavior (Akpoghiran et al., 2024). Mated females show decreased sleep and lower rhythmicity strength, potentially also influencing seizure behavior. In our study, we did not deliberately select for virgins; females were collected when 0 to 16 h old. As such, a large majority would have been virgin, but perhaps not all. Thus, we cannot state that mating status influenced seizure severity.

Seizure severity of *jus* females followed a bimodal circadian rhythm, with two peaks and two troughs across the 24 h. In the context of *Drosophila*, the daily locomotor activity rhythm also follows a bimodal circadian rhythm, as shown by the circadian locomotor activity results of this study. Activity peaks at lights-on, ZT 0 termed “morning peak,” and at lights-off, ZT 12 termed “evening peak” (Miyasako et al., 2007; Chiu et al., 2010), precisely match the seizure severity peaks observed in *jus* female mutants at ZT 12, and close to ZT 24 (= ZT 0). PDF-positive s-LNvs are integral pacemakers for *Drosophila* circadian locomotor activity and play a major role in driving morning peaks of activity (Miyasako et al., 2007), as well as in the regulation of the phase of other cells, such as pre-motor neurons (Liang et al., 2019). Peptides released by central pacemaker neurons (e.g., DH44 and Hugin) link the clock to motor outputs (King et al., 2017), thus modulating the locomotor activity rhythm. Increased synaptic excitation of motoneurons is associated with seizure activity in *jus* mutants (Marley and Baines, 2011). Hence, potentially, increased PDF-positive s-LNvs neural activity and excitability could underlie seizure peaks: thus, seizure burden following a bimodal circadian rhythm similar to that of locomotor activity. In addition, the vortex assay induces seizures by overstimulating *Drosophila* sensory bristles (Kuebler and Tanouye, 2000). Circadian regulation of sensory inputs, such as circadian rhythms in light sensitivity (Vinayak et al., 2013), could be involved in driving the observed seizure severity rhythm. To further substantiate the proposed bimodal circadian rhythm observed in BS females, a pharmacologically induced seizure model could be assessed. Wild-type flies fed a proconvulsant (Stilwell et al., 2006) could be tested at different times of the day, thus examining the reproducibility of the bimodal rhythm in alternative seizure models.

Having established a potential influence of time of day on seizure severity of *jus* females, this study sought to ablate the central clock to determine its role in seizure rhythmicity. Under constant illumination, *Drosophila* become arrhythmic due to constant degradation of dCRY upon photoactivation (Konopka et al., 1989; Peschel et al., 2009). Hence, the molecular clock becomes arrhythmic. BS females maintained in LL conditions showed no oscillation in seizure severity, indicative of an underlying contribution from circadian physiology.

Although constant light abolished seizure rhythmicity, photic masking could be involved in *jus* seizure activity. Circadian rhythms can be “masked” by other stimuli presented at predictable times of day (Gall and Shuboni-Mulligan, 2022), such as light, causing the direct enhancement or inhibition of locomotor activity (Lamont and Amir, 2009). To discriminate whether seizure rhythmicity of *jus* females is circadian-mediated or masked by light, flies were reared under free-running DD conditions. Results from this suggested an effect of CT on seizure severity despite the absence of light stimuli, implying an effect of circadian rhythms on seizure severity of *jus* females. Studies have shown that *Drosophila* free-running circadian rhythms in DD remain stable and persist with a period close to 24 h (Plautz et al., 1997; Johnstone et al., 2022), similar to the circadian locomotor results in this study. Moreover, *jus* females maintained in free-running DD conditions exhibited a seizure severity rhythm with a longer period than in LD, thus seizure peaks were ~4 h later in the day. Regarding the locomotor activity of *jus* females monitored in DD, this also seemed to be shifted relative to the LD rhythm. Under DD conditions, the “siesta” or inactivity period in the middle of the respective day is less pronounced (Mazzotta et al., 2020). Hence, locomotor activity in DD did not show two peaks of activity like seizure severity, thus not being able to match locomotor activity to seizure severity profiles in DD. Therefore, arguments for increased activity and nervous system excitability leading to increases in seizure severity cannot be extended to DD as in LD.

While environmental manipulations (lighting conditions) implicate the circadian clock in seizure severity of *jus* females, this study also benefited from direct genetic evidence. Incorporating the *per^0^* mutant to the *jus* background resulted in the absence of a rhythm in seizure recovery time, thus providing strong causal support that seizure rhythmicity is driven by the molecular clock. Furthermore, immunohistochemistry revealed normal PER oscillations in four of the main clock neuron groups; hence, we can establish that the molecular oscillator is intact in the *jus* background.

In conclusion, this study provides evidence for an interplay between circadian rhythms and *Drosophila* seizure activity: a barely researched phenomenon. Our findings suggest a potential bimodal circadian rhythm of seizure severity in female seizure mutants (*jus* and *para^bss1^*). Given that *Drosophila* is well suited for investigating the relationship between circadian rhythms and seizure activity, future work should focus on the mechanism(s) modulating seizure rhythmicity downstream of clock genes. Excitingly, an enhanced understanding could lead to the targeting and prevention of seizures for the better treatment of epilepsy.

## Supplemental Material

sj-docx-1-jbr-10.1177_07487304261428337 – Supplemental material for Circadian Rhythms Time Seizure Severity in Drosophila Supplemental material, sj-docx-1-jbr-10.1177_07487304261428337 for Circadian Rhythms Time Seizure Severity in Drosophila by Mariam Huertas-Radi, Adam A. Bradlaugh and Richard A. Baines in Journal of Biological Rhythms
