# Supplementary material for: Circadian Rhythms Time Seizure Severity in Drosophila
Source: J Biol Rhythms. 2026 Apr 9;41(4):400–15. doi: 10.1177/07487304261428337 (PMC13342475; doi:10.1177/07487304261428337)
Supplement: sj-docx-1-jbr-10.1177_07487304261428337 – Supplemental material for Circadian Rhythms Time Seizure Severity in Drosophila [file sj-docx-1-jbr-10.1177_07487304261428337.docx]

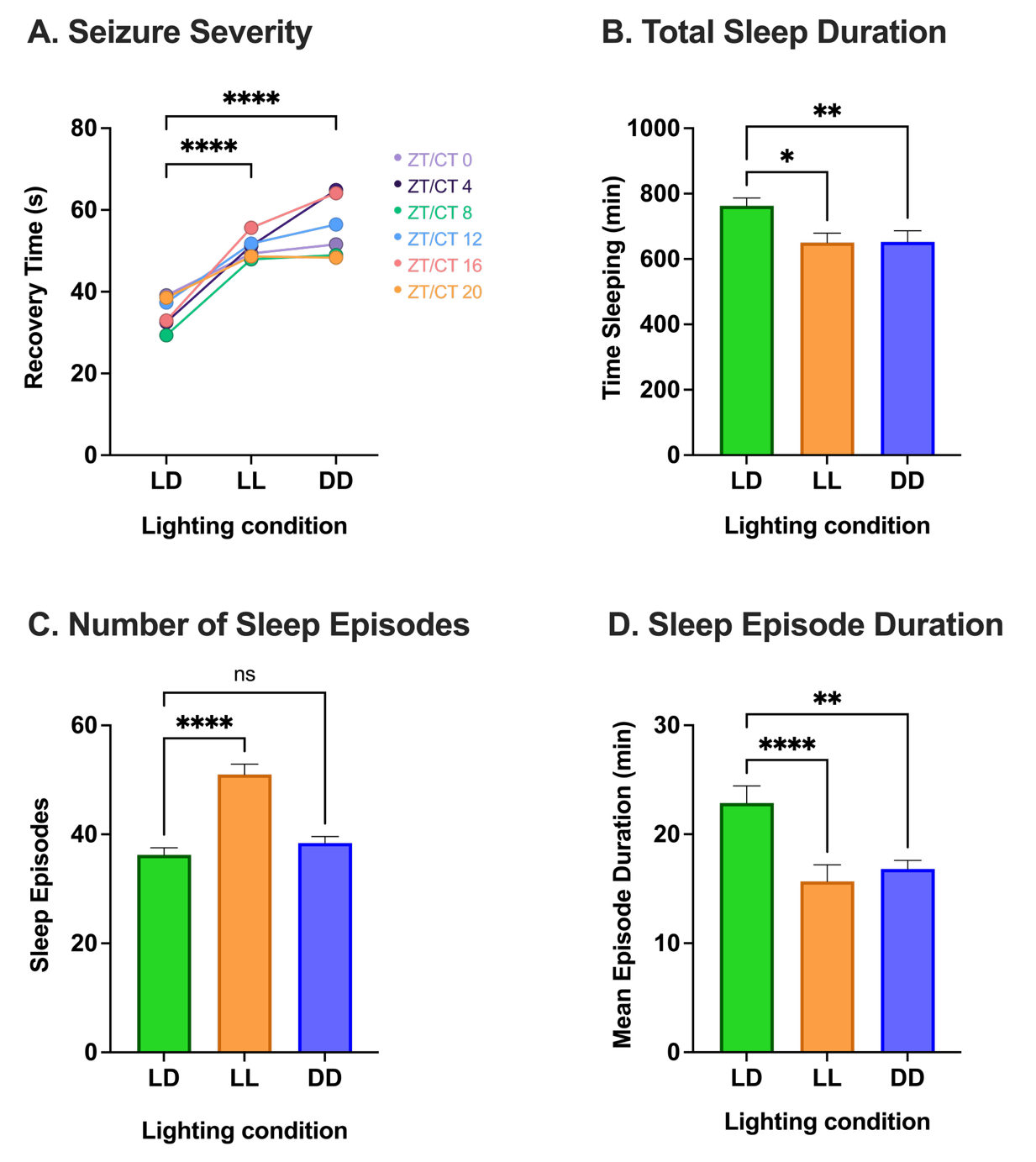
**Supplementary material**

**Figure S1. Constant light (LL) and constant darkness (DD) show an increase in seizure severity in *jus* females, potentially linked to sleep loss and fragmentation.** (**A**) Lighting condition has a statistically significant effect on seizure severity of *jus* females tested via the vortex assay. Seizure recovery times in LL and DD show an increase in seizure severity compared to 12:12 LD conditions at all timepoints (two-way ANOVA, *F* (2, 719) = 111, *p* < 0.0001, n = 6 timepoints). Full descriptive statistics and *p* values of multiple comparison tests are reported in Table S1. Each dot represents the mean seizure recovery time for each timepoint, per lighting condition. Timepoints are coloured differently and joined across lighting conditions with lines. LD vortex data is taken from Fig. 1B, LL from Fig. 2A, and DD from Fig. 2C. (**B**) Total sleep duration of *jus* females reared in LL (in orange) and DD (in blue) was significantly lower than in LD (in green) (Kruskal-Wallis test*, H* (2) = 11.87, *p* < 0.05 and *p* < 0.01, respectively, n = 25-28). (**C**) Number of sleep episodes of *jus* females monitored in LL, but not in DD, were significantly higher than in LD (one-way ANOVA, *F* = 28.5, *p* < 0.0001 and *p* = 0.47, respectively, n = 25-28). (**D**) Duration of sleep episodes of *jus* females kept in LL and DD were significantly lower than in LD (Kruskal-Wallis test, *H* (2) = 28.05, *p* < 0.0001 and *p* < 0.01, respectively, n = 25-28). Statistically significant comparisons from Dunnett and Dunn’s multiple comparison tests are indicated as **** *p* ≤ 0.0001, *** *p* ≤ 0.001, ** *p* ≤ 0.01, * *p* ≤ 0.05. Full descriptive statistics and *p* values of multiple comparison tests are reported in Table S2. Data are presented as mean ± SEM.

**Table S1. Full descriptive statistics of multiple comparison tests of seizure severity in LL and DD compared to control LD conditions of *jus* females**

| Timepoint (ZT/CT) | Conditions compared | Mean recovery time (s) | SEM | post hoc test | *p* value | Significance | Fig. |
| --- | --- | --- | --- | --- | --- | --- | --- |
| 0 | LD  LL  DD | 39.10  49.37  51.61 | 1.57  2.17  3.69 | Dunnett’s  Dunnett’s | < 0.01  < 0.001 | **  *** | 4A |
| 4 | LD  LL  DD | 32.58  51.24  64.89 | 0.96  2.59  4.62 | Dunnett’s  Dunnett’s | < 0.0001  < 0.0001 | ****  **** | 4A |
| 8 | LD  LL  DD | 29.33  47.93  48.95 | 0.94  2.47  2.32 | Dunnett’s  Dunnett’s | < 0.0001  < 0.0001 | ****  **** | 4A |
| 12 | LD  LL  DD | 37.35  51.78  56.44 | 1.35  1.85  2.76 | Dunnett’s  Dunnett’s | < 0.0001  < 0.0001 | ****  **** | 4A |
| 16 | LD  LL  DD | 32.95  55.65  64.1 | 0.90  2.78  4.16 | Dunnett’s  Dunnett’s | < 0.0001  < 0.0001 | ****  **** | 4A |
| 20 | LD  LL  DD | 38.54  48.65  48.35 | 1.48  1.54  2.24 | Dunnett’s  Dunnett’s | < 0.05  < 0.05 | *  * | 4A |

**Table S2. Full descriptive statistics of multiple comparison tests of sleep parameters in LL and DD compared to control LD conditions of *jus* females**

| Sleep parameter | Conditions compared | Mean | SEM | post hoc test | *p* value | Significance | Fig. |
| --- | --- | --- | --- | --- | --- | --- | --- |
| Total Sleep Duration (min) | LD  LL  DD | 763.0  650.2  652.2 | 23.77  28.79  34.52 | Dunn’s  Dunn’s | < 0.05  < 0.01 | *  ** | 4B |
| Number of Sleep Episodes | LD  LL  DD | 36.24  51.0  38.42 | 1.30  1.90  1.19 | Dunn’s  Dunn’s | < 0.0001  = 0.47 | ****  ns | 4C |
| Sleep Episode Duration (min) | LD  LL  DD | 22.87  15.67  16.81 | 1.58  0.80  1.53 | Dunn’s  Dunn’s | < 0.0001  < 0.01 | ****  ** | 4D |

**
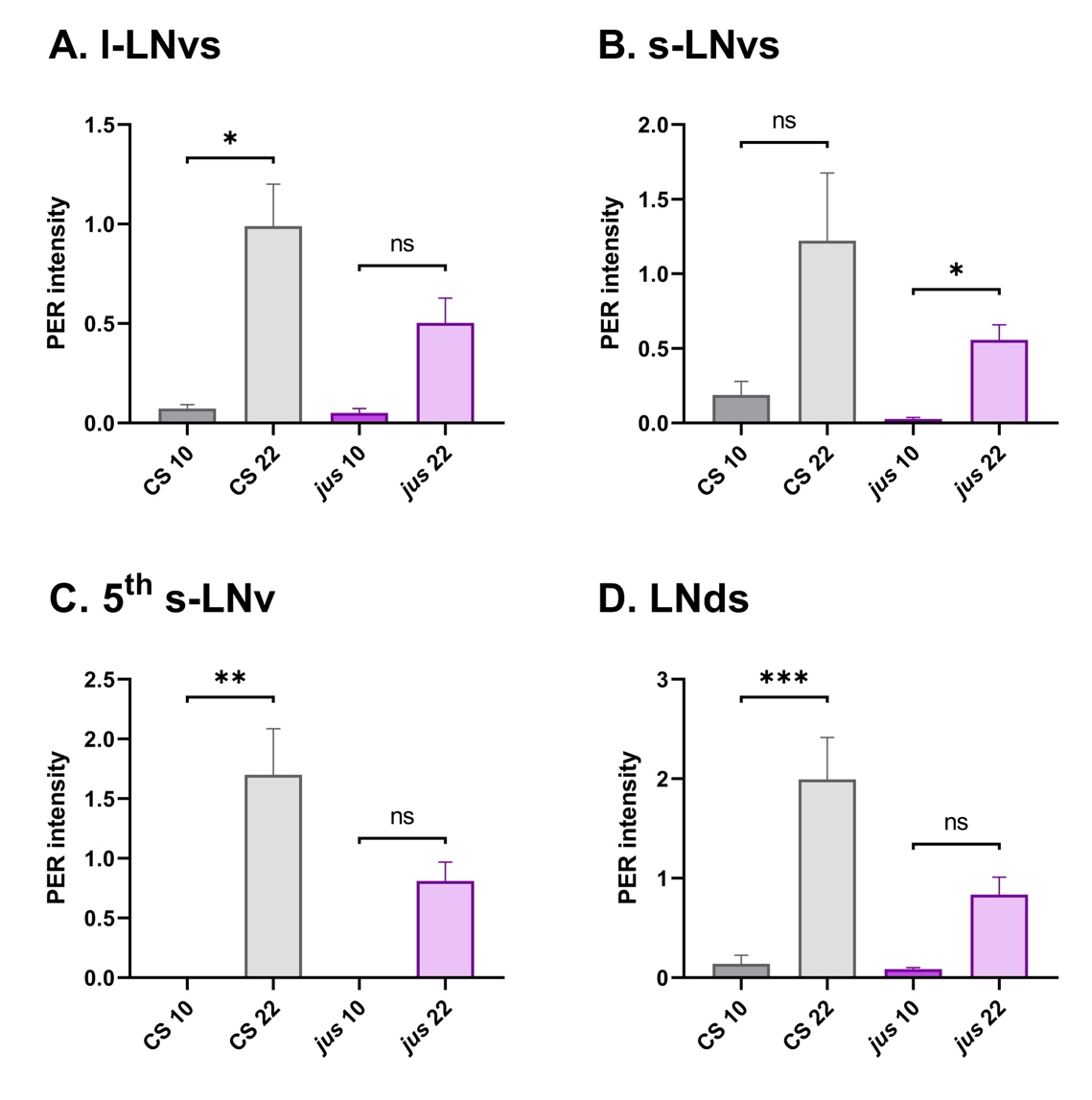
**

**Figure S2. *Jus* males show trending, but not significant, PER oscillations in clock neurons.** The intensity of PER expression was quantified from antibody staining of *jus* (purple) and CS control (grey) adult male fly brains, in four clock neuron groups: large ventral-lateral neurons (l-LNvs, **A**), small ventral-lateral neurons (s-LNvs, **B**), the 5^th^ s-LNv (**C**), and dorsal-lateral neurons (LNds, **D**). CS males showed statistically significant differences between PER intensity at ZT 10 and 22 in l-LNvs (Kruskal-Wallis test with Dunn’s multiple comparison test, *p* < 0.05), 5^th^ s-LNv (Kruskal-Wallis test with Dunn’s multiple comparison test, *p* < 0.01) and LNds (one-way ANOVA with Tukey’s multiple comparison test, *p* < 0.001). By contrast, *jus* males showed significant differences only in s-LNvs (Kruskal-Wallis test with Dunn’s multiple comparison test, *p* < 0.05). Statistically significant comparisons from Dunn’s and Tukey’s multiple comparison tests are indicated as *** *p* ≤ 0.001, ** *p* ≤ 0.01, * *p* ≤ 0.05. Clock neurons from five brain hemispheres were quantified for each condition. Quantification was normalised to background. Data are presented as mean ± SEM.
